# A social network analysis: mental health scales used during the COVID-19 pandemic

**DOI:** 10.3389/fpsyt.2023.1199906

**Published:** 2023-08-29

**Authors:** Shuang Chen, Xue Lan, Han Yu

**Affiliations:** Department of Health Management, China Medical University, Shenyang, China

**Keywords:** COVID-19, mental health, scale, named entity recognition, social network analysis

## Abstract

**Introduction:**

The focus on psychological issues during COVID-19 has led to the development of large surveys that involve the use of mental health scales. Numerous mental health measurements are available; choosing the appropriate measurement is crucial.

**Methods:**

A rule-based named entity recognition was used to recognize entities of mental health scales that occur in the articles from PubMed. The co-occurrence networks of mental health scales and Medical Subject Headings (MeSH) terms were constructed by Gephi.

**Results:**

Five types of MeSH terms were filtered, including research objects, research topics, research methods, countries/regions, and factors. Seventy-eight mental health scales were discovered.

**Discussion:**

The findings provide insights on the scales used most often during the pandemic, the key instruments used to measure healthcare workers’ physical and mental health, the scales most often utilized for assessing maternal mental health, the tools used most commonly for assessing older adults’ psychological resilience and loneliness, and new COVID-19 mental health scales. Future studies may use these findings as a guiding reference and compass.

## 1. Introduction

The new coronavirus disease 2019 (COVID-19), caused by the SARS-CoV-2 virus, was declared a pandemic following an increasing number of cases worldwide (1). COVID-19 is characterized by serious illness presentations such as respiratory symptoms leading to acute respiratory distress syndrome (ARDS), cardiovascular abnormalities, multiple organ failure, septic shock, and death (2, 3). Apart from physical medical consequences, it may have a significant impact on mental health and well-being (4).

Previous studies suggest that individuals infected with SARS-CoV-2 develop sadness and anxiety, which are precursors or risk factors for suicidality (5). COVID-19 patients experience a high rate of impaired awareness, disorientation, and post-traumatic stress disorder (PTSD) (6). A recent review of the mental health outcomes of quarantine and similar prevention strategies discovered that depression, anxiety disorders, mood disorders, posttraumatic stress symptoms, sleep disorders, panic, stigmatization, low self-esteem, and lack of self-control are all common among people who have been subjected to physical isolation (7). Another rapid evaluation found that stressors such as protracted quarantine, fear of infection, frustration, boredom, insufficient supplies, insufficient information, financial loss, and stigma caused PTSD, bewilderment, and rage in the general population (8). COVID-19 causes waves of heightened dread and anxiety, which are known to cause significant changes in the behavior and psychological well-being of many people.

The focus on psychological issues during COVID-19 has led to the development of large surveys that involve the use of mental health scales. A wide variety of mental health measurements are available, including both existing anxiety or depression scales, such as the Self-Rating Anxiety Scale (SAS) and the Hospital Anxiety and Depression Scale (HADS), and those developed in response to COVID-19, such as the Coronavirus Anxiety Scale (CAS). In a study conducted by Lei et al. (9), mental health status was assessed using the SAS and the Self-Rating Depression Scale (SDS). Another cross-sectional study by Liang et al. (10) used the General Health Questionnaire (GHQ-12), Negative Coping Styles Scale, and PTSD Checklist-Civilian Version (PCL-C) to examine mental health in juveniles. A comparable study used the Patient Health Questionnaire (PHQ) and the Generalized Anxiety Disorder (GAD-7) to measure depression and anxiety (11). Fountoulakis et al. (12) published COMET-G study including a large sample size from worldwide. They used the cut-off score 23/24 for the Center for Epidemiological Studies-Depression (CES-D) Scale and a derived algorithm to identify cases of probable depression, the STAI-S, the State Anxiety Scale (S-Anxiety) of the State–Trait Anxiety Inventory (STAI) and the Development of the Risk Assessment Suicidality Scale (RASS) to assess anxiety and suicidality, respectively.

Additionally, several studies were carried out to investigate the prevalence of mental health among healthcare practitioners during COVID-19. A single-center cross-sectional survey utilized a Numeric Rating Scale (NRS) on fear, the Hamilton Anxiety Scale (HAMA), and the Hamilton Depression Scale (HAMD) to assess the mental health of healthcare personnel and administrative employees (13). An online survey used the Impact of Event Scale (IES), GAD-7, PHQ to monitor psychological distress in hospital staff (14). Clearly, the options for mental health scales are extensive.

The selection of mental health measurements varies according to the object, topic, method, and region of the study. Thus, determining how to choose the appropriate measurement is of great concern. Researchers usually resort to exploring a large amount of literature to screen for appropriate and efficient measurement tools, which is undoubtedly laborious. To address this problem, scholars have used methods, such as systematic reviews, to summarize the mental health measurements used within particular research contexts. For instance, Smarr and Keefer (15) compiled several measurement tools that are relevant to the assessment of depression in rheumatic research. Balsamo et al. (16) focused on self-report surveys that are regularly and presently used to measure anxiety in older individuals. Lazor et al. (17) reviewed anxiety measurements quantitatively in children or adolescents with cancer or undergoing hematopoietic stem cell transplantation. While these reviews are useful resources, their content is often too specialized to be applied in other contexts.

In this work, text mining was employed to automatically extract information on mental health scales to comprehensively characterize them. By applying algorithmic, statistical, and data management methods to the vast amount of knowledge existing in unstructured texts, text mining enables researchers to identify the needed information more efficiently, uncover relationships hidden in the sheer volume of available information, and generally shift the burden of information overload from the researchers to the computer (18). In recent years, researchers have begun to investigate how text mining techniques can be used to process medical data. Lucini et al. (19) used text mining methods to process data from patient records of early emergency departments. Other study interests pertaining to medical information processing using text mining approaches include obesity event mining (20), sexual event mining (21), and smoking event mining (22).

To the best of our knowledge, no prior research has focused on the text mining of mental health scales. Therefore, this research uses text mining and social network analysis to identify mental health instruments used during COVID-19 and examines the application features of the measures. This study addressed the following questions: (1) Which mental health scales were most often utilized during COVID-19? (2) What are the various scales’ research objectives, research objects, and research methods? (3) Has there been any variation in the nations and areas where the scale is used? (4) What scales are utilized for health workers, pregnant women, and the older adults? and (5) What is the name of the new COVID-19 mental health scale?

## 2. Materials and methods

### 2.1. Research design

**Figure 1:**
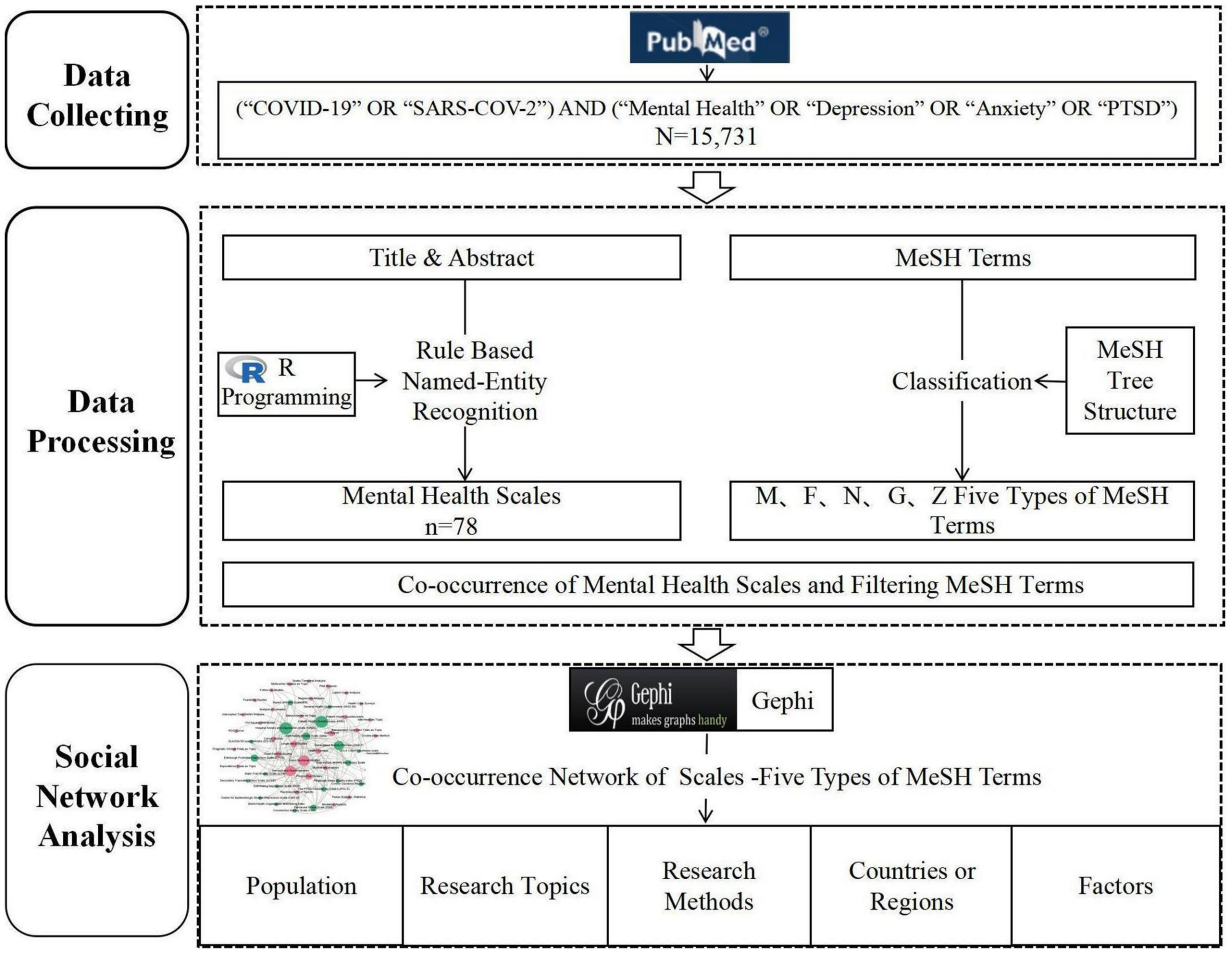
Framework diagram.

In this study, the social networks based on the co-occurrence of mental health scales and MeSH terms were created to analyze the characteristics of the application of mental health scales during COVID-19. The research design framework is illustrated in Figure 1. Specifically, the following series of steps were completed: (1) retrieving relevant research articles from the PubMed database; (2) collecting information on the titles, abstracts, Medical Subject Headings (MeSH) terms, and PMIDs of the articles retrieved from PubMed; (3) extracting the entities of mental health measurement from the titles and abstracts; (4) filtering MeSH terms with specific semantic types; and (5) constructing the network through the co-occurrence of the entities of mental health measurements and filtering MeSH terms using Gephi 0.9.2 software.

### 2.2. Information sources

For the following reasons, the PubMed database was chosen as the data source. PubMed database is a biomedicine database produced and managed by the National Library of Medicine (NLM), with broad coverage, rapid updates, and a comprehensive retrieval mechanism. PubMed contains standardized bibliographic information to aid in the extraction of fields such as article titles, abstracts, and MeSH terms, and tools for extracting and processing PubMed data in bulk are available. The MeSH word list is a manually normalized dynamic narrative word list, which not only standardizes the near-synonyms and different expression forms of the same concept, but also provides a tree structure (Tree Number) to help users understand the semantic relationships and subordination among subject words, and facilitates the selection of subject words of specific semantic types from them for describing the application of mental health scales.

The articles’ raw data were retrieved on October 28, 2021 [query = (“COVID-19” OR “SARS-COV-2″) AND (“Mental Health” OR “Depression” OR “Anxiety” OR “PTSD”)] by PubMed. 15,731 publications were identified and incorporated. The titles, abstracts, PMIDs, and MeSH terms were downloaded and stored in PubMed format.

### 2.3. Data processing

The research was designed to extract the entities of mental health scales and their applications. The network was used to present the association between those entities. This study’s proposed approach is explained, as follows.

First, Named Entity Recognition (NER) was performed. NER is fundamental in several natural language processing applications. It entails locating and classifying text into predetermined categories such as a person’s name and location. In this study, a rule-based approach was used to recognize entities of mental health scales that occur in the text. Rule-based techniques need a vocabulary of proper names and a collection of patterns (23), as the naming of mental health scales has a certain pattern, such as starting with capital letters, containing the word “scale,” “instrument,” “tool,” and so on. For the NER process, R Studio, an integrated development environment (IDE) for the R programming language, was employed. It provides a user-friendly interface and a range of tools that facilitate data analysis and text processing. The capabilities of R Studio were leveraged to extract mental health scales from the titles and abstracts of research articles. The identified mental health scales were cleaned and the names were standardized by four researchers in two separate groups. For example, the Depression, Anxiety and Stress Scale was written in DASS, Depression, Anxiety and Stress Scale-21 or DASS-21. They were expressed uniformly as “Depression, Anxiety and Stress Scale (DASS-21).” After normalization, a lexicon of mental health scales was formed to facilitate subsequent data extraction for large samples.

Second, MeSH terms were extracted and classified. The MeSH tree structure was employed to discriminate between different semantic types. MeSH tree numbers are assigned to each MeSH term and organized into 16 categories: anatomical words are in category A, biological phrases are in category B, disease keywords are in category C, and so on. A MeSH tree structure allows MeSH concepts belonging to different semantic types to be classified using specific descriptors and numbers (24). Combining the purpose of the study and the categories of the MeSH word list, MeSH words for population (M), study method (N05 and N06.850.520), region (Z), mental health (F), and phenomenological process (G) were extracted by R-studio.

Finally, Network analysis and visualization were employed to construct a network based on the co-occurrence of the scales and MeSH terms in the same article. The approach of network analysis arose from computer science to demonstrate the impact of social networks. The network was built using the Gephi 0.9.2. It is a widely used open-source software specifically designed for network analysis and visualization. And it offers a user-friendly interface and various tools for network exploration and analysis. In the co-occurrence networks of the scales and MeSH terms, the nodes represent the entities of mental health scales and five types of MeSH terms including research objects, research topics, research methods, countries/regions, and factors, and the linkages that reflect the frequency with which these entities co-occur. The betweenness centrality of each node was computed to better comprehend the relationships between these entities. The frequency with which a node contacts the geodesic paths of other network nodes yields the betweenness centrality, which shows a node’s significance in a network (25).

## 3. Results

### 3.1. Summary characteristics of the included scales

According to the above-mentioned search strategy, 15,731 articles were included in this analysis. After extracting scales from the titles and abstracts of the articles, removing duplicates, and using inclusion and exclusion criteria, 78 mental health scales were identified (Multimedia Appendix 1). Table 1 lists the 20 scales with the most occurrences. The scales covered anxiety, depression, sleep disturbance, well-being index, and so on. Scale ranking was based on the frequency of their appearance in the titles and abstracts of mental health studies during the COVID-19 pandemic. The most frequent scales were the PHQ, HADS, GAD-7, DASS-21, and IES. Most scales were used repeatedly, with four scales used more than 100 times and 14 scales used only once.

**Table 1:** The most occurrences scales (*N* = 20).

No.	Scale	Numbers	Percentage (%)
1	Patient Health Questionnaire (PHQ)	177	15.94
2	Hospital Anxiety and Depression Scale (HADS)	130	11.71
3	Generalized Anxiety Disorder (GAD)	118	10.63
4	Depression, Anxiety and Stress Scale (DASS-21)	102	9.18
5	Impact of Event Scale(IES)	57	5.13
6	Self-Rating Anxiety Scale (SAS)	55	4.95
7	State–Trait Anxiety Scale (STAI)	41	3.69
8	Perceived Stress Scale (PSS)	36	3.24
9	Edinburgh Postnatal Depression Scale (EPDS)	23	2.07
10	Connor Davidson Resilience Scale (CD-RISC)	23	2.07
11	Pittsburgh Sleep Quality Index (PSQI)	23	2.07
12	UCLA 3-Item Loneliness scale	22	1.98
13	The PTSD Checklist for DSM-5 (PCL-5)	19	1.71
14	World Health Organization Well-Being Index	18	1.62
15	Coronavirus Anxiety Scale (CAS)	13	1.17
16	Self-Rating Depression Scale (SDS)	12	1.08
17	Secondary Traumatic Stress Scale (STSS)	11	0.99
18	Center for Epidemiologic Studies Depression Scale (CES-D)	10	0.90
19	General Health Questionnaire (GHQ-28)	10	0.90
20	EuroQol-5D questionnaire (EQ-5D)	10	0.90

### 3.2. Mental health scales in different research objects

**Figure 2:**
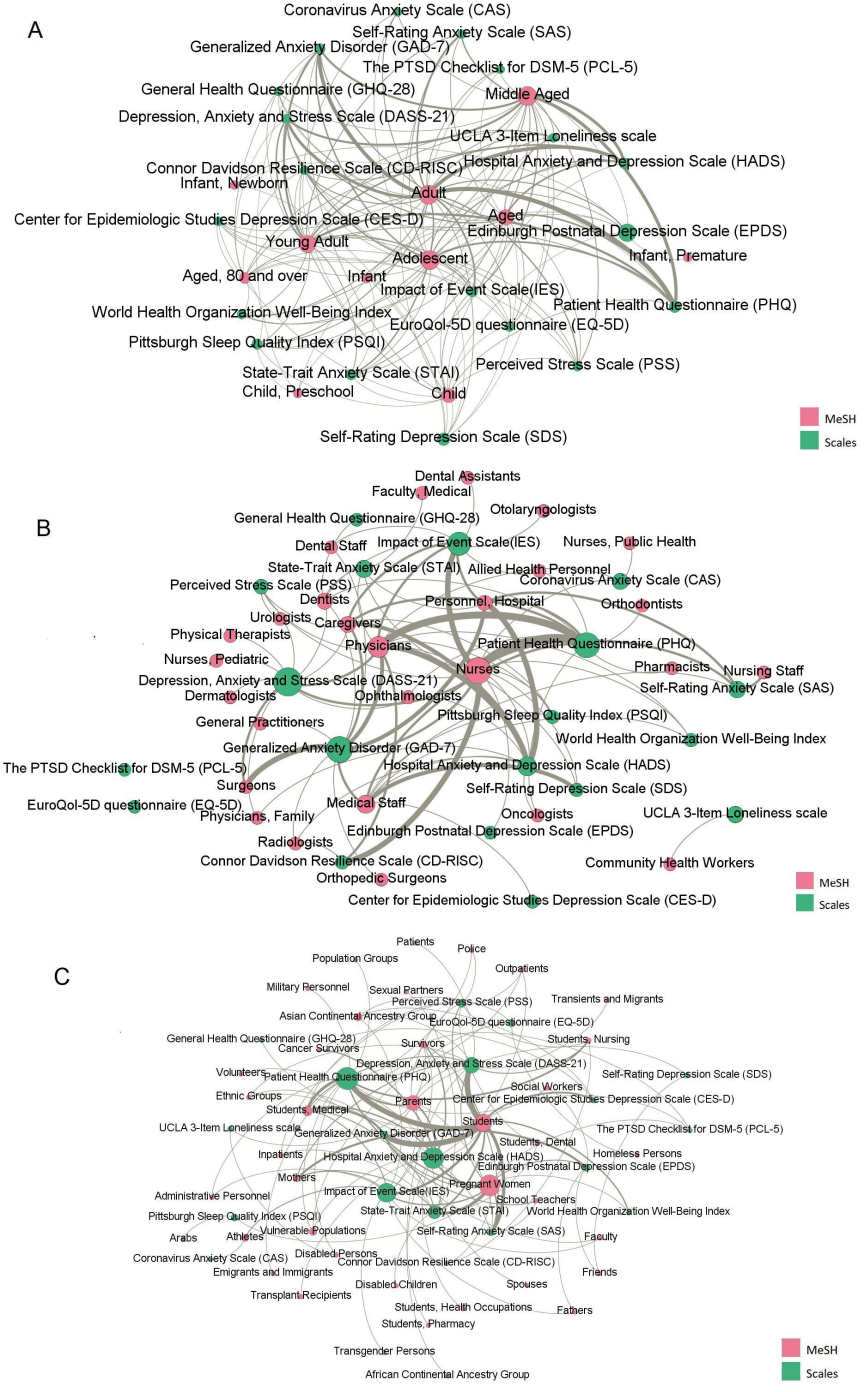
The co-occurrence network of scales and Medical Subject Headings (MeSH) terms representing the research population. **(A)** The co-occurrence network between scales and MeSH terms representing various age groups. **(B)** The co-occurrence network between scales and MeSH terms representing health personnel. **(C)** The co-occurrence network between scales and MeSH terms representing other groups of the population, excluding various age groups and health personnel. Pink labels represent scales, and green labels represent the MeSH terms. The edges represent their co-occurrence relationships.

The co-occurrence network graphs between the scales and various age groups (Figure 2A), health personnel (Figure 2B), and other groups (Figure 2C) were constructed based on the application of the scales in different groups to reveal the application characteristics of the scales with various research objects.

Most of the study participants were adults and young adults, who had the highest frequency and betweenness centrality among the other age groups. Additionally, Figure 2A indicates that the Edinburgh Postnatal Depression Scale (EPDS) is significantly related to the MeSH terms “Infant, Newborn” and “Infant,” which mainly focused on the mental health status of postpartum women during the COVID-19 pandemic, including whether COVID-19 will aggravate postpartum depression and the relationship between the feeding behavior of premature infants’ mothers and postpartum depression during the pandemic. Furthermore, the impact of isolation measures during COVID-19 on maternal breastfeeding behavior and mental health was examined. Second, the mental health status of older adults during the COVID-19 pandemic was a widespread concern. The most used scales were the PHQ and HADS, which aimed to explore COVID-19 prevention policies, such as Turkey’s curfew policy (26), and the declining mental health of the older adults caused by isolation (27).

Nurses received the most attention when health professionals were considered as the research objects. The most used scale was the PHQ, followed by the Connor Davidson Resilience Scale (CD-RISC) and IES. When investigating the mental health status of physicians, the HADS and PHQ were used widely.

Additionally, students and pregnant women have also received widespread research attention. The PHQ, DASS-21, and GAD-7 were mainly used to detect the mental health status of college students, especially medical students during COVID-19; the STAI, HADS, and SAS were widely used to detect the health problems of pregnant women during COVID-19, such as anxiety, depression and psychological stress.

### 3.3. Research topics of mental health scales

**Figure 3:**
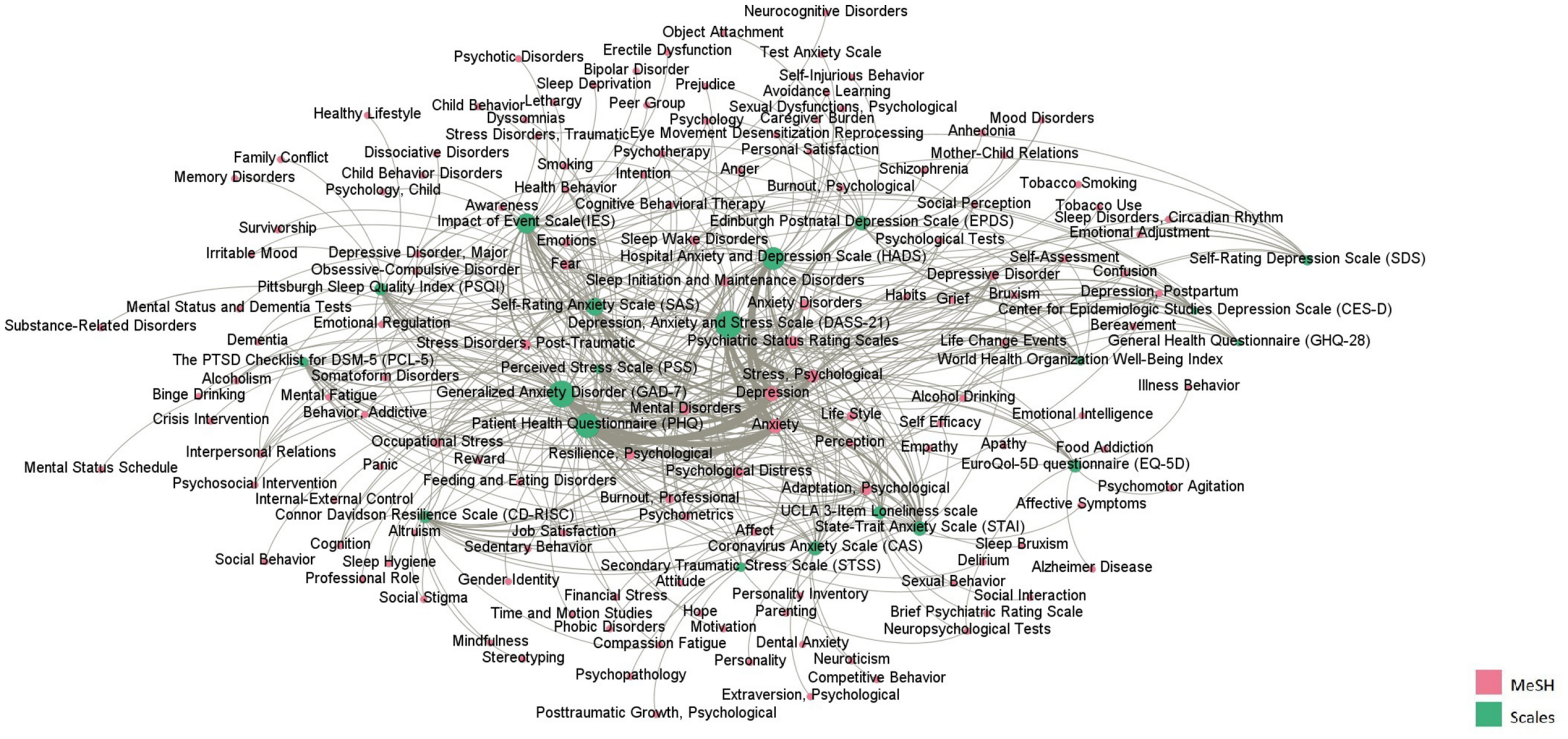
The co-occurrence network between scales and MeSH terms representing research topics.

The co-occurrence network graph of the scales and mental health MeSH terms (Figure 3) reflects the application of the scales to different mental health problems. Depression, anxiety, and stress were the most common research topics. As shown in Figure 3, the PHQ had the highest betweenness centrality in all scales, followed by the DASS-21, GAD-7, HADS, and IES. They were most widely used in the field of mental health. The PHQ was the most used instrument to study anxiety and depression, usually used with the Insomnia Severity Index (ISI). The DASS-21 was usually used to analyze psychological stress, which was used frequently with the IES. The CD-RISC was mostly used to study resilience, perception, cognition, and job satisfaction.

### 3.4. Research methods of mental health scales

**Figure 4:**
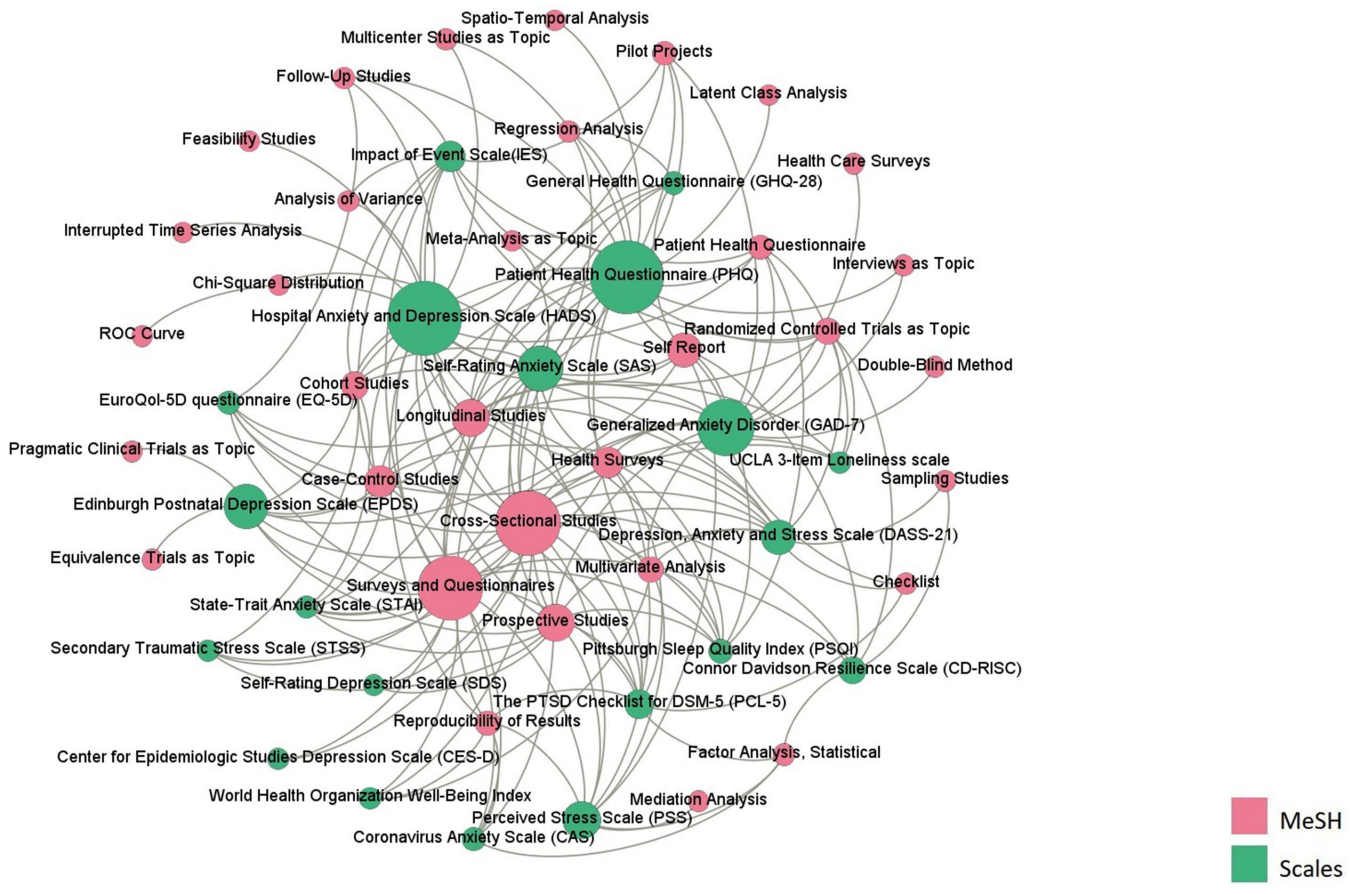
The co-occurrence network between scales and MeSH terms representing research methods.

As shown in Figure 4, the HADS had the largest betweenness centrality, followed by the PHQ, GAD-7, DASS-21, and IES. The most used research method was cross-sectional study (*N* = 20), followed by longitudinal research (*N* = 13) and prospective study (*N* = 12). The HADS was the most used instrument in prospective study and longitudinal research.

### 3.5. Application of mental health scales in different countries/regions

**Figure 5:**
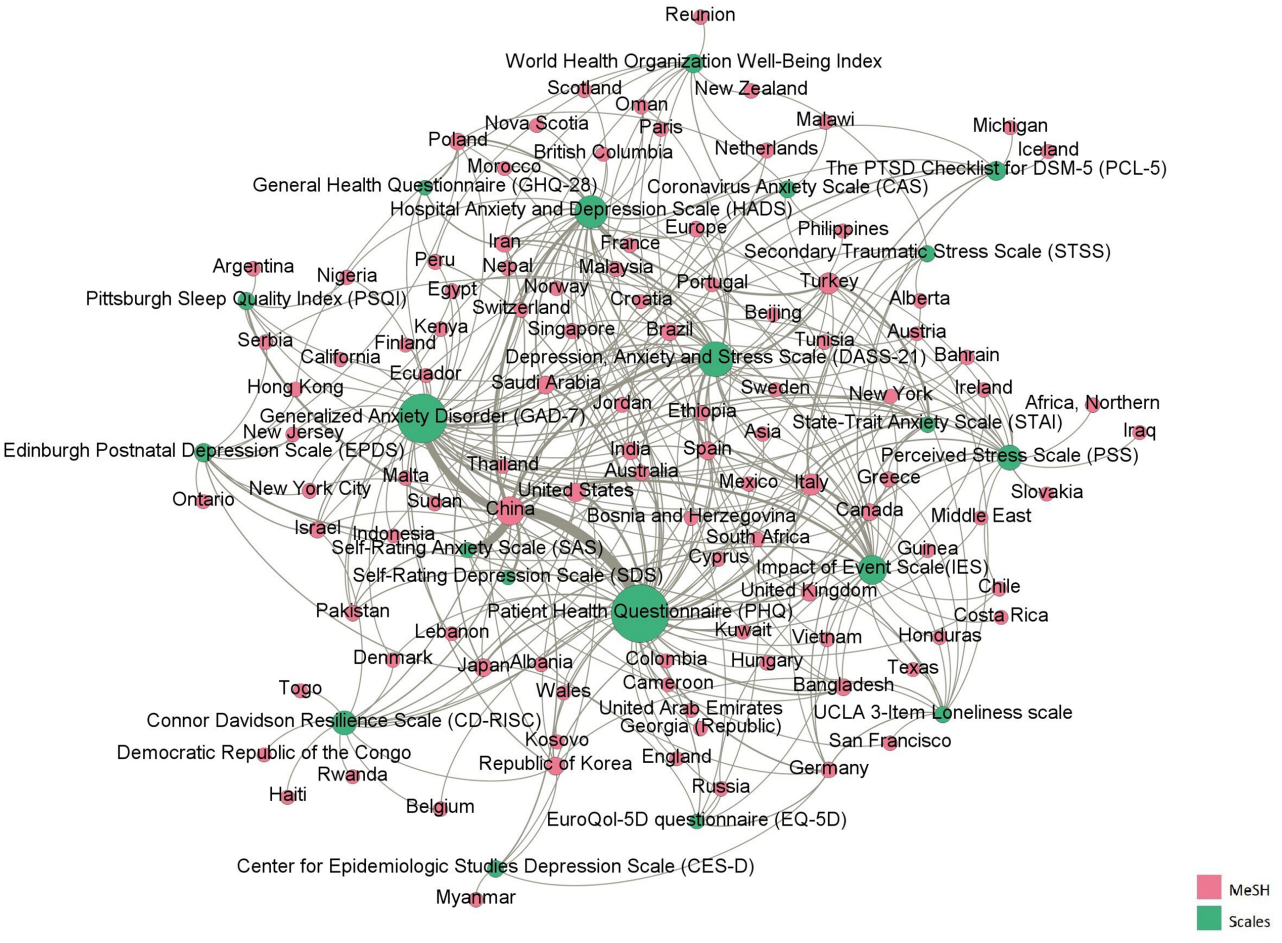
The co-occurrence network between scales and MeSH terms representing countries/regions.

The mental health scales were used in more than 113 countries/regions during the COVID-19 pandemic. As shown in Figure 5, China has the largest betweenness centrality, which means the studies were conducted mostly in China and that it had the greatest influence. The three scales that co-occurred most frequently with China were the PHQ, SAS and GAD-7. This was followed by Italy, and the commonly used scales were the IES and PHQ. In Turkey, the commonly used scales were the STAI and HADS.

### 3.6. Factors in mental health research

**Figure 6:**
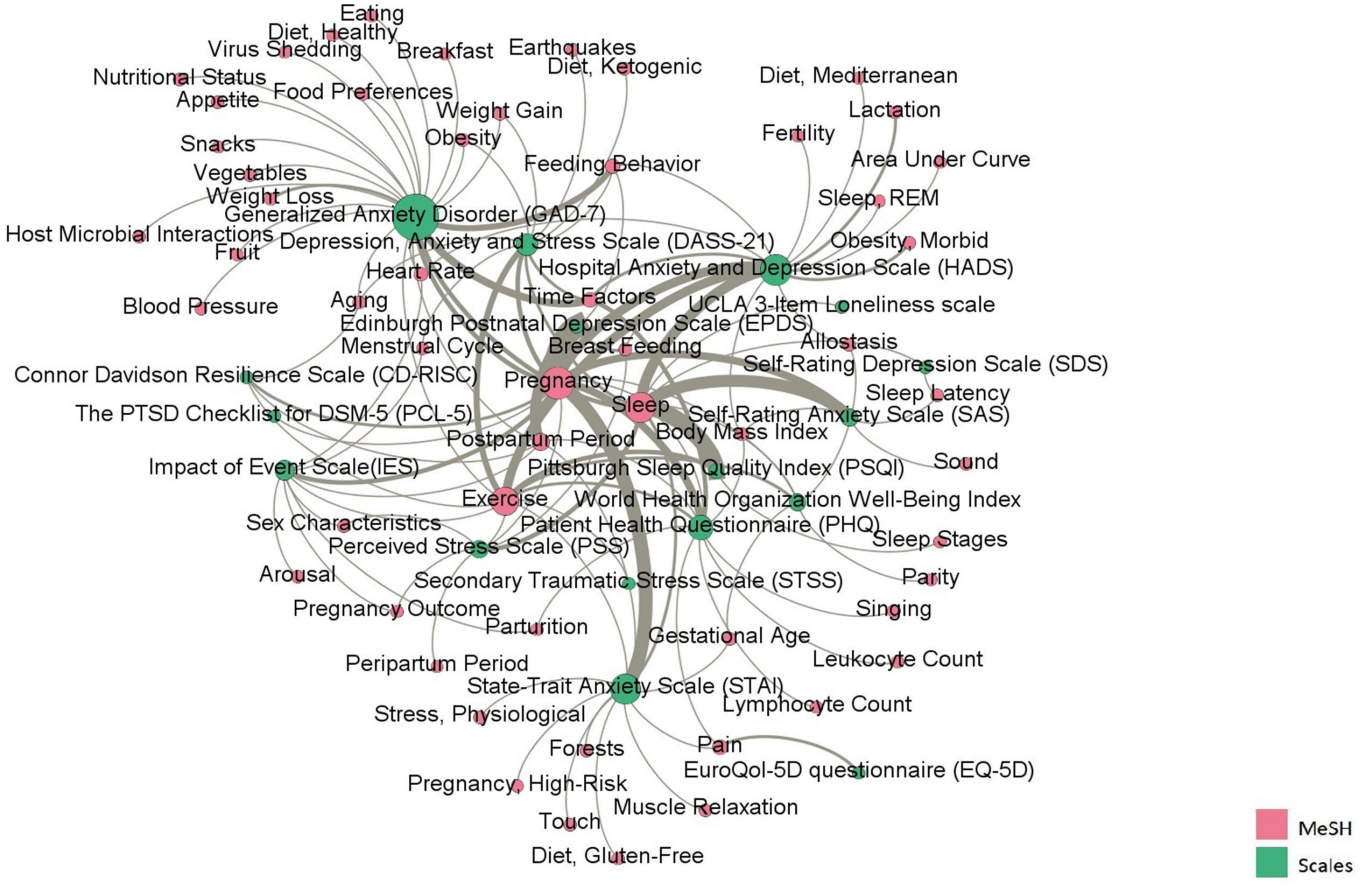
The co-occurrence network between scales and MeSH terms representing factors.

Figure 6 reflects the factors concerned in the research during COVID-19. The MeSH terms were sorted according to the betweenness centrality. The MeSH terms with the most occurrences included pregnancy, sleep, and exercise, which are involved in the application of most scales. The scales closely related to pregnancy include the EPDS, STAI, and PHQ; the scales closely related to sleep were the PSQI, and SAS; the scales closely related to exercise were the HADS and DASS-21. The scale with the largest betweenness centrality was the GAD-7. In addition to focusing on the above phenomena, the GAD-7 has also commonly been used to assess the impact of fear and anxiety on nutrition during the COVID-19 pandemic, mainly involving MeSH terms in nutritional physiology (e.g., fruits, snacks, and nutrition).

## 4. Discussion

### 4.1. Most widely used scales

Based on the results of the social network analysis, the PHQ was found to be the most commonly used mental health scale during the pandemic, followed by the HADS, GAD-7, DASS-21, and IES. Using the aforementioned measures, there is a high degree of consistency in the selection of study participants, including persons of varying ages and medical practitioners. In Table 2, the MeSH terms that appear most often in conjunction with the five instruments were summarized.

**Table 2:** Five scales and high-frequency co-occurrence Medical Subject Headings (MeSH) terms.

Scale	Population	Countries	Research methods	Factors
PHQ	Adult	China	Cross-sectional studies	Pregnancy
	Health Personnel	Italy	—	Time Factors
	Adolescent	United States	—	Exercise
HADS	Adult	China	Cross-sectional studies	Sleep
	Aged	Turkey	Prospective studies	Exercise
	Adolescent	France	Cohort studies	Pregnancy
GAD	Adult	China	Cross-sectional studies	Time Factors
	Adolescent	United States	Longitudinal studies	Feeding Behavior
	Aged	Saudi Arabia	—	Pregnancy
	—	—	—	Sleep
DASS-21	Adult	China	Cross-sectional studies	Exercise
	Adolescent	Spain	Longitudinal studies	Pregnancy
	Aged	Brazil	Prospective studies	Sleep
	—	—	—	Time factors
IES	Adult	China	Cross-sectional studies	Pregnancy
	Adolescent	Italy	Longitudinal studies	—
	Health personnel	Spain	Pilot projects	—

This study found that the PHQ-9 was used more frequently, applicable to detect depression, anxiety, and psychological stress across a diverse group of patients, including adults, adolescents, older adults, health professionals, the disabled, and severely ill patients. Additionally, the countries with the most applications of the PHQ-9 were China (28–30), Italy (31, 32), and the United States (33, 34).

The HADS has been used extensively and applied to all age groups and participants with different identities. As it is specific to medical settings; has good psychometric properties; facilitates brief, rapid administration; and has good compliance, most studies using the HADS have been conducted on a sample of patients with diseases, such as cancer (35), Parkinson’s disease, or other chronic diseases (36). Regarding the status of mental health, this study found that the HADS united other instruments such as the IES and ISI to study psychological distress (37–39), psychological stress (40, 41), and job burnout (39, 42) among healthcare workers and patients.

The GAD-7 is now one of the most extensively used anxiety measures, both in clinical practice and research, due to excellent diagnostic reliability and factorial, construct, and criterion validity (43, 44). The GAD-7 was usually used to examine the relationship between work-related fatigue and mental health among healthcare workers exposed to COVID-19 (45, 46). Additionally, the countries with the most applications of the GAD-7 were China (47–49), the United States (50, 51), and Saudi Arabia (52, 53).

The DASS-21 is a widely used screening tool designed to measure depression, anxiety, and stress (54). Australia (55), the United States (56), and England (54) are among the English-speaking nations that have provided proof of the DASS-21’s validity for usage in clinical and community settings. This study found that the DASS-21 was mainly used to measure adults, adolescents, and older adults in China, Spain, and Brazil during COVID-19.

The IES was developed by Mardi Horowitz et al. (57) to measure current subjective pain associated with a specific event. IES is probably the most widely used self-report measure in the field of traumatic stress (58). It played a crucial role in assessing the psychological impact of the COVID-19 on various groups, with a focus on evaluating anxiety, psychological stress, and depression among the target population, including the general population, healthcare workers and pregnant women.

In conclusion, social network analysis provides valuable insights into the prevalence and usage patterns of different mental health scales during the COVID-19. The findings highlight the importance of these tools in assessing depression, anxiety, stress, and psychological distress across different groups. The widespread adoption of these scales highlights their relevance and reliability in various cultural and linguistic contexts, making them valuable instruments for mental health assessment, especially during challenging times like the COVID-19 pandemic.

However, it is crucial to recognize that while these scales are widely used, they should not replace comprehensive clinical assessment and professional judgment. A comprehensive understanding of an individual’s mental health requires an integrated approach that considers both quantitative assessments from the scale and qualitative insights from a skilled mental health professional. By integrating these components, mental health practitioners can provide a more accurate and comprehensive assessment, leading to the right interventions and support for individuals experiencing mental health challenges.

### 4.2. Scales for healthcare personnel

With many new cases of depression, anxiety, physical and mental exhaustion, stress, and burnout as well as recurrences of previously diagnosed cases (59–61), this global health crisis has put a strain on the entire healthcare system and jeopardized the wellbeing of frontline healthcare workers. Depression, burnout, resilience, sleep quality, secondary traumatic stress (STS), and PTSD were the main health topics among healthcare personnel during the COVID-19 (62). Most studies employed the Maslach Burnout Inventory (MBI) as their primary tool for evaluating burnout (63–65)， while the most popular tool for evaluating the psychological resilience of healthcare professionals was the CD-RISC. The PSQI was the most used sale to evaluate issues or occurrences linked to sleep. These instruments were used together to analyze the relation between psychological disorder and the exposure to COVID-19.

Providing direct care to traumatized populations is now recognized as carrying a risk of STS. The Secondary Traumatic Stress Scale (STSS) is a 17-item questionnaire used to assess the detrimental effects of indirect exposure to traumatic events in healthcare professionals who are caring for distressed or traumatized patients (66). During COVID-19, the STSS was used to assess the STS state of health personnel in Turkey (67, 68), India ((69)), and the Republic of Cyprus (70). The PTSD checklist for DSM-5 (PCL-5) is a 20-item self-report test that assesses PTSD symptoms on a five-point Likert scale during the last month (71). Studies found that in the COVID-19 period, a considerable proportion of health personnel experienced PTSD (72).

### 4.3. Scales for pregnant women

When compared to pregnant women examined before the COVID-19 pandemic, pregnant women assessed during the pandemic reported higher discomfort and mental health symptoms, mostly in the form of depression and anxiety symptoms (73). Although there were various scales used for measuring the degree of anxiety and depression during the pandemic, the EPDS and STAI have been used most commonly to assess maternal mental health. The EPDS, developed by Cox et al. (74), which assesses the severity of postpartum depression experienced by women. It was widely used during COVID-19 to assess the depressive symptoms of pregnant and postpartum women (75, 76).

The STAI is frequently employed for the anxiety screening process. This measurement has two subscales, the State Anxiety Scale (S-Anxiety) and the Trait Anxiety Scale (T-Anxiety). The STAI was mainly used to screen for the presence and severity of state and trait anxiety of pregnant women during the COVID-19 pandemic.

### 4.4. Scales for older adults

Early in the pandemic, older people were recognized as a risk category for mental health consequences, since older age was soon established as the primary risk factor for severe and fatal COVID-19 prognoses (30). Further to concentrating on the older adults’ despair and anxiety, it is important to consider their resilience and loneliness. The CD-RISC and UCLA 3-Item Loneliness (UCLA-3) scale were the most used instruments for measuring psychological resilience and loneliness among older adults. The number of items in these two instruments are 10 and 3, which is convenient for respondents to fill in, especially older adults. Moreover, the UCLA-3 was mainly applied in the United States, Bangladesh, and Germany, and was not widely used in China during the COVID-19 pandemic.

### 4.5. Scales for COVID-19

To address a gap in the mental health response to this escalating public health catastrophe, researchers created a set of mental health screeners that may be used to accurately detect likely instances of dysfunctional anxiety and the severity of symptoms related to COVID-19, including the CAS, Corona Disease Anxiety Scale (CDAS), COVID-19 Peritraumatic Distress Index (CPDI), COVID-19 Stress Scale (CSS), and Fear of COVID-19 Scale (FCV-19S).

The CAS was created and released by (77). in March 2020, in response to the pandemic’s demands for a quick assessment of COVID-19 phobia. The seven items of the CAS must be answered on the Likert scale of 1–5. The overall index, which is calculated by summing the scores for each item, runs from 7 to 35 points; the higher the score, the greater the COVID-19 dread. It can be used to identify probable cases of dysfunctional anxiety associated with the coronavirus. Currently, the CAS is translated into Portuguese (78), Korean (79), Bangla (80), Arabic (81), Turkish (82), Japanese (83), Cuban (84), Polish (85), Spanish (86), Chinese (87), and Brazilian (88).

Alipour et al. (89) created the CDAS in the Farsi language. This measurement includes 18 questions and four-point Likert answers. Higher CDAS scores imply a high level of anxiety caused by COVID-19. Cronbach’s alpha value of 0.919 was used in the original investigation to establish the scale’s reliability.

The CPDI is an online tool that was created in China during the COVID-19 epidemic (6). It is probably the most complete measure of COVID-related psychological discomfort, as it includes symptoms from a wide range of mental health disorders and diseases. This tool evaluates COVID-19 peritraumatic distress symptoms through a self-report questionnaire with 24 items. It also sought information on demographics, the frequency of anxiety, depression, particular phobias, cognitive shifts, avoidance and compulsive behavior, physical symptoms, and loss of social functioning over the previous 2 weeks (90). The CPDI has been used in several countries, such as India (91), Iran (92), and Germany (93).

The preceptors’ discomfort in relation to COVID-19 was assessed using the 36-item CSS (94). The self-reporting tool consists of five subscales, including traumatic stress symptoms, obsessive checking, disease-related xenophobia, risk and contamination worries, and socioeconomic effects. Respondents were asked to rate each symptom on a five-point Likert scale. It was used in South Korea (95), Canada (96), and Singapore (97).

The FCV-19S (98) was established in 2022, which was not published at the time of data acquisition in this study. In order to ensure the comprehensiveness of the scales collection, it was considered to be included and introduced. The seven-item FCV-19S was often utilized during COVID-19 and exhibited trustworthy psychometric characteristics. It is valid and trustworthy in measuring COVID-19 fear among the general public.

## 5. Limitations

Two main limitations of this study must be acknowledged. First, in terms of data selection, PubMed was chosen above alternative databases such as the Web of Science Core Collection. This research concentrated on journals and paid little attention to other forms of scientific information transmission (such as books, working papers, and reports). Consequently, some significant studies, particularly developing research, may have been overlooked.

Second, MeSH terms were used to extract terms of research objects, research topics, research methods, countries/regions, and factors. However, MeSH terms are mostly composed of commonly used ideas, excluding new concepts. To address this limitation, deep learning techniques will be utilized in subsequent experiments to extract entities from the complete text and reduce the effort associated with human annotation.

## 6. Conclusion

Seventy-eight mental health scales were successfully identified by named entity recognition. Based on the co-occurrence networks of scales-research objects, scales-research topics, scales-research methods, scales-countries/regions, and scales-factors, the following findings were found: (1) the PHQ, DASS-21, HADS, GAD-7 and IES were most often utilized during COVID-19; (2) depression, anxiety, and stress are the most common research topics; (3) the most commonly used research method was cross-sectional study, followed by longitudinal research and prospective studies; (4) the studies were conducted mostly in China, and the three scales mostly used in China were the PHQ, SAS and GAD-7; (5) the MBI, CD-RISC, PSQI, STSS, and PCL-5 were the instruments mainly used to assess the burnout, resilience, sleep quality, STS, and PTSD of healthcare professionals; (6) the EPDS and STAI were the scales mostly used for maternal mental health; (7) the CD-RISC and UCLA-3 were the most used instruments for measuring psychological resilience and loneliness among older adults; and (8) the CAS, CDAS, CPDI, CSS, and FCV-19S are the new COVID-19 mental health scales.

This study aimed to provide researchers with a comprehensive view of the various facets of mental health scales during COVID-19 and also emphasized the characteristics of these scales when applied. The results of this study may help the researchers choose the appropriate scale efficiently, without reading a large amount of literature to screen the scale. Future research in this area may use above findings as a crucial reference and compass.

## Data availability statement

The original contributions presented in the study are included in the article/supplementary material, further inquiries can be directed to the corresponding author.

## Author contributions

XL: conceptualization. XL and SC: methodology, formal analysis, and writing—review and editing. XL and HY: software. HY, XL, and SC: validation. SC: writing—original draft preparation and visualization. All authors have read and agreed to the published version of the manuscript.

## Conflict of interest

The authors declare that the research was conducted in the absence of any commercial or financial relationships that could be construed as a potential conflict of interest.

## Publisher’s note

All claims expressed in this article are solely those of the authors and do not necessarily represent those of their affiliated organizations, or those of the publisher, the editors and the reviewers. Any product that may be evaluated in this article, or claim that may be made by its manufacturer, is not guaranteed or endorsed by the publisher.

## Supplementary material

The Supplementary material for this article can befound online at: https://www.frontiersin.org/articles/10.3389/fpsyt.2023.1199906/full#supplementary-material

Appendix 1 List of Identified Mental Health Scales (*N*=78).
